# Editorial: New advances in our understanding of fertilization

**DOI:** 10.3389/fvets.2022.1043407

**Published:** 2022-10-14

**Authors:** Alejandro Vicente-Carrillo, Adriana Casao, Manuel Álvarez-Rodríguez

**Affiliations:** ^1^Ciencianova, Investigación y Desarrollo Magapor A.I.E. Zaragoza, Zaragoza, Spain; ^2^Grupo BIOFITER, Departamento de Bioquímica y Biología Molecular y Celular, Facultad de Veterinaria, Instituto Universitario de Investigación en Ciencias Ambientales de Aragón, Universidad de Zaragoza, Zaragoza, Spain; ^3^Department of Animal Reproduction, Instituto Nacional de Innovación Agraria - Consejo Superior de Investigaciones Científicas (INIA-CSIC), Madrid, Spain

**Keywords:** spermatozoa, oocyte, fertilization, cattle, pig, dog

Spermatozoa are highly reactive cells that, on their way to the oocyte, interact with a series of cells and fluids in order to overcome the pre-fertilization and fertilization process. All these interactions are going to produce cell responses in spermatozoa as well as cellular and molecular changes in the female genital tract. Those cell- and molecular responses are in charge of preparing the female genital tract for future pregnancy and, in turn, getting the oocyte ready prior to fertilization. There is still a lack of knowledge on the sperm-female genital tract and sperm-egg interactions and sperm-egg interactions, as well as embryo development. Through a wide variety of analytical tools such as light and electron microscopy, flow cytometry, transcriptomics, etc., it is possible to investigate the mechanisms of such interactions and thus increase our knowledge of the pre-fertilization and fertilization processes. Thus, elucidating the molecular and cellular mechanisms that spermatozoa, the oocyte, and the female genital tract overcome prior to, and during, fertilization is critical for our understanding of fertilization.

In the first paper on this Research Topic, Son et al. aimed to improve *in vitro* embryo production by analyzing the effect of superstimulation in Holstein cows located in the Gulf area, at high temperatures. Superovulation induction with an intramuscular injection of pregnant mare serum gonadotropin (PMSG) on day 14 of the estrus cycle. 40 h after the PMSG injection, the oocytes were collected. Among the parameters studied, the number of follicles with a diameter higher than 6 mm and the number of retrieved cumulus-oocyte complexes, the maturation, cleavage, and blastocyst formation rates after somatic cell nuclear transfer were significantly higher in the superstimulated group than in the control group. Therefore, a single injection of PMSG could improve the field conditions for reaching an efficient production of cloned cow embryos.

In the second paper, Imakawa et al. elaborate a comprehensive review trying to elucidate the reasons behind the high failure of the assisted reproductive techniques (50% in cattle and up to 75% in human fertilized eggs/blastocysts), particularly relevant during the first 3–4 weeks of pregnancy. Both blastocyst hatching and implantation to the maternal endometrium proceeds are concomitant with a large cohort of physiological events such as epithelial-mesenchymal transition (EMT) and trophoblast cell fusion. Moreover, it has been recently described the pivotal role of extracellular vesicles (EVs) with micro RNAs (miRNAs) and long non-coding RNAs (lncRNAs) in the establishment of the proper uterine environment required for peri-implantation processes. The authors included, in this comprehensive review, new and ongoing research on EVs, miRNA, and lncRNAs in order to elucidate their plausible connections with conceptus implantation to the maternal endometrium.

Two papers focused on reproductive functions in the porcine model follow this Research Topic. In the paper of Ausejo et al., the authors set up a comparative experiment of three different techniques: sperm chromatin stability assay (SCSA), terminal deoxynucleotidyl transferase dUTP nick end labeling (TUNEL) assay, and sperm chromatin dispersion test (SCD, Halomax^®^). The results showed a statistically significant correlation only between SCSA and TUNEL data, whereas total or progressive sperm motility was not correlated with sperm nuclear DNA fragmentation (sDF). In contrast, there was a positive correlation between TUNEL measure and abnormal acrosomes (%) and between SCD measure and total sperm morphological abnormalities (%). However, no significant correlations were obtained between fertility or prolificacy results and sDF results with the different techniques. Nevertheless, and being aware that the different techniques for the sDF do not target the same DNA events, the studied sDF techniques could be a valuable tool for studying sperm quality parameters through non-classical analytical methods and, thus, could be explored as useful for the management of artificial insemination procedures in the farms.

Shi et al. paper explore ELF4 effects during zygotic gene activation (ZGA). In this study, early porcine embryos and tissues ELF4 expression were analyzed. ELF4 knock-outs were used to analyze the changes in H3K9me3 modification, DNA methylation, and ZGA-related genes. Their results showed the presence of ELF4 expression at all stages of early porcine embryos fertilized *in vitro* (IVF), being the highest expression level at the 8-cell stage. The ELF4 knock-out induced an increase in DNA damage at the 4-cell stage caused by the decrease of both embryonic developmental competency and blastocyst quality. Moreover, it was an abnormal increase in H3K9me3 and DNA methylation levels at the 4-cell stage and inhibited the expression of genes related to ZGA, caused by interfering with ELF4. Thus, these results suggest that ELF4 affects ZGA and embryonic development competency in porcine embryos by maintaining genome integrity, regulating dynamic changes of H3K9me3 and DNA methylation, and correctly activating ZGA-related genes to promote epigenetic reprogramming. Overall, these results provide a theoretical basis for further studies on the regulatory mechanisms of ELF4 in porcine embryos.

This Research Topic ends up with the scientific research conducted by Mitjana et al. on how photoperiod and melatonin supplementation affects the quality of chilled dog semen. Based on a potential seasonal effect caused by photoperiod duration, the hypothesis tested laid on the addition of a physiological concentration of melatonin in the canine ejaculate. Melatonin had no effect on motility in either photoperiod. However, some adverse effects of melatonin were detected in acrosomal defects, apoptosis, and viability in the decreasing photoperiod. Moreover, the addition of melatonin to sperm in the decreasing photoperiod could create such a high level that it could be linked to the detected negative effects. In contrast, a beneficial effect of melatonin in the increasing photoperiod on acrosomal defects and apoptosis was found. Moreover, in the short term (days 1 and 2) melatonin treatment increased the viability for both photoperiods. Finally, in the medium term (days 2 and 3), melatonin can benefit mitochondrial activity but only in decreasing photoperiod.

From sperm quality assessment, photoperiod and heat influence in gamete development, and molecular physiology of embryos, the results of the above-mentioned studies and reviews offered a representative scientific piece of new relevant data on the understanding of fertilization. Despite being clear literature and evidence related to this critical topic, the papers published in this Research Topic show that there is still room to study many aspects, particularly at the level of gamete quality assessment, *in vivo* and *in vitro* embryo development and the effect of climate change in animal breeding. All of these studies would contribute to improve the overall understanding of the complex topic of animal reproduction in our constantly changing world.

## Author contributions

AV-C, AC, and MA-R contributed to writing, reviewing, and editing the current manuscript. All authors approved the final version of this editorial.

## Conflict of interest

The authors declare that the research was conducted in the absence of any commercial or financial relationships that could be construed as a potential conflict of interest.

## Publisher's note

All claims expressed in this article are solely those of the authors and do not necessarily represent those of their affiliated organizations, or those of the publisher, the editors and the reviewers. Any product that may be evaluated in this article, or claim that may be made by its manufacturer, is not guaranteed or endorsed by the publisher.

